# The interpretation of [+distal] in demonstratives and complementizers

**DOI:** 10.1515/ling-2022-0178

**Published:** 2023-08-04

**Authors:** Camil Staps, Johan Rooryck

**Affiliations:** Leiden University and Radboud University Nijmegen, Nijmegen, The Netherlands; cOAlition S and Leiden University, Leiden, The Netherlands

**Keywords:** Common Ground, exclamative *that*, main clause *que*/*că*, optional *that*, proximal/distal

## Abstract

This article argues that the [+distal] feature of demonstrative *that* is also present in complementizer *that*, and has not bleached away. In particular, we argue that complementizer *that* is referential: it refers to an element in the Shared Discourse Space (an extension of the Common Ground) that can be seen as distal. This allows us to explain (i) that direct speech patterns with [−distal] (*Sue said this*/#*that: “It is raining”*) while indirect speech patterns with [+distal] (*Sue said* *this/*that it is raining*); (ii) the use of *that* in exclamatives (*That bio industry is still allowed!*); and (iii) that optional *that* is more frequently used when there is some sort of context between Speaker and Addressee. This last phenomenon has parallels in Romance complementizers derived from Latin *quod*, which can likewise be seen as [+distal]. We propose that [+distal] is a marker of Addressee involvement, which can account for all these phenomena, and can be extended to demonstrative uses of *that*. In exophoric contexts, [+distal] additionally marks actual distance. The interpretation of Addressee involvement and actual distance depends on context; we propose that it is derived from the interaction between the syntactic DP/CP domain and the pragmatic exophoric/endophoric distinction.

## 1 Introduction

Indirect speech reports are commonly formed by a verb of saying and a finite complementizer introducing the sentential complement. In English, as in many other languages, this complementizer developed from a distal demonstrative, and it is indeed not possible to use a form with a proximal feature in this position. This suggests a close association between indirect speech reports and [+distal]:

(1)

**Table:** 

Sue said (*this/that) it is raining.
(cf. Rooryck 2019: 257)

The opposite is the case with direct speech reports. Direct speech is usually introduced only by a pause, or by quotation marks in writing (*Sue said: “It is raining”*). However, the speech report can be referred to with a cataphoric pronoun in the main clause.1Whether direct speech reports are subordinated or paratactic structures is inconsequential to our argument. This pronoun is then necessarily [−distal]:2As an anonymous reviewer points out, *that* is possible in (2) if used anaphorically rather than cataphorically, e.g. *But he has said that: “Am I supposed to dislike them?”*. In such cases *that* is not coindexed with the speech report as *this* can be; instead, *that* refers to something in the previous context between Speaker and Addressee. We leave these cases out of consideration here.

(2)

**Table:** 

*Sue said (this/#that): “It is raining.”*
(cf. Rooryck 2019: 257)

How can we explain the relationship between direct/indirect speech and [−/+distal], respectively? Of course *that* in (1) and *this* in (2) have a different syntactic category, but that is irrelevant to our question since we are comparing the value of the [±distal] feature that *this* and *that* have in common. Simply claiming that the complementizer *that* is semantically bleached and entirely lacks a [±distal] feature is not sufficient; this simply shifts the question to the history of the form: why did *that*, and not *this*, develop into a finite complementizer (cf. Kayne 2014: 189)? Instead, we present a new, unified analysis which predicts a broad range of ways in which the proximal/distal distinction is recycled in both demonstrative and complementation environments, explaining the contrast in (1)–(2) as well as many other data adduced below.

The standard view on complementizers like *that* in (1) is that they fulfill a primarily syntactic function and are largely void in terms of semantics and pragmatics, apart from carrying a feature indicating that they introduce a tensed rather than an untensed complement clause (Lasnik and Saito 1991: 324; Rizzi 1997: 312; implicitly in Rosenbaum 1965 and various grammars, e.g. Huddleston and Pullum 2002: 947–1030; and see discussion in Roberts and Roussou 2003: 111–116). In other words, mainstream theories of complementation do not attribute any synchronic value to the original distal semantics of the complementizer *that*, nor do they ascribe any other interpretively relevant information to it. However, a number of studies have indicated that these complementizers do carry additional interpretive information (e.g. Bolinger 1972; Dor 2005; Storms 1966; Yaguchi 2001). So far, such studies have mostly been restricted to rather specific contexts. Below, we will first draw attention to a number of recurring interpretive properties of complementizers and sketch the outline for a unified account. In this way, our account of the difference between *this* and *that* in (1)–(2) will also allow us to explain the difference between zero and *that* in English object clauses (*I thought* (*that*) *you might need some help*), the use of overt complementizers in exclamatives (*That bio industry is still allowed!*), and evidential interpretations of root complementizer constructions in Romance (to be exemplified below). We then show how these recurring properties can be explained as the interpretive recycling of a [±distal] feature. This allows for a general analysis of *this* and *that* covering both demonstrative and complementizer functions.

Concretely, we will argue that the proximal/distal distinction inherent in demonstratives can be recycled in two different ways, which we call actual distance and Addressee involvement.3For the term recycling, see Rooryck (2019: 244), building on Biberauer (2017). What we mean by this is that markers of a certain category (here, proximal/distal) are repurposed to mark features of a different category (here, actual distance and Addressee involvement). This may be the first step in a grammaticalization process, in which the original deictic meaning has not been lost (yet). This perspective on *that* is thus quite different from the traditional view, which takes demonstratives and complementizers as *de facto* homonyms, at least synchronically (e.g. Diessel 1999: 123–125). It yields a more economical, polysemous view of demonstratives as exercising chameleon-like, distinct but strongly related functions, that vary according to the syntactic and pragmatic context in which they are used. The interpretation of these categories differs depending on the context. The overall picture that we are working towards is as in Table 1. On the left we have demonstratives, which reference entities in the DP domain. These can be exophoric or endophoric (Diessel 1999: 93–100). Exophoric demonstratives refer to something in the speech situation (*this*/*that book*), while anaphoric demonstratives refer indirectly through a linguistic antecedent in the surrounding discourse ([*Sales have been going up*]_i_. [*This trend*]_i_ …). The complementizer *that* plays a role in the CP domain. We see it as referring to information content as opposed to entities in the speech situation. This reference can still be exophoric (when it refers to a concrete utterance, e.g. *Sue said that it is raining*) or anaphoric (when it refers indirectly through the Speaker’s model of the discourse state, as in *I thought that you might need some help*; Bolinger 1972: 58).

**Table 1:** Deriving different kinds of reference from two binary properties.

	Entities (DP)	Information content (CP)
Exophoric	Exophoric demonstratives (Section 4):	Direct/indirect speech (Section 2):
	Actual distance in the concrete physical world	Actual distance in a multidimensional conceptual world, interpreted as similarity
	Addressee involvement: interpreted as psychological factors (psychological distance, joint attention, empathy, …)	Addressee involvement: interpreted as evidentiality; proximity is private witness evidentiality
Anaphoric	Anaphoric demonstratives (Section 5):	Presupposition (Section 3):
	Addressee involvement: *that* used over *this* to interact and empathize with the Addressee	Addressee involvement: *that* used over zero to signal content in the Shared Discourse Space

We begin our discussion with reference to information content. In Section 2, we use direct and indirect speech reports to introduce the notions of actual distance and Addressee involvement. Actual distance reflects the similarity between the speech report and the original utterance, whereas Addressee involvement is related to evidentiality. For the interpretation of Addressee involvement we introduce an extension of the notion of Common Ground, which we call Shared Discourse Space. The Shared Discourse Space, unlike the Common Ground, includes not only common commitments to propositions, but in broad terms all entities and information content that are jointly tracked by Speaker and Addressee as part of the discourse context (see Section 2 below for a more precise definition). Roughly, a direct speech report is more similar to the reported utterance than an indirect speech report (actual distance), and an indirect speech report places Speaker and Addressee on an equal footing with respect to the evidence for the reported utterance (Addressee involvement). We then move on to complementizers more generally in Section 3, showing how Addressee involvement can explain alternations between overt and zero complementizers in a variety of environments (e.g., exclamative *that*, as in *That bio industry is still allowed!*, marks a presupposition that is shared with the Addressee). Sections 4 and 5 are dedicated to showing that the proximal/distal distinction is used in a similar way in demonstratives. Here, actual distance is simply physical distance to the object pointed at (*this*/*that book* being close to or far from the Speaker, respectively), and Addressee involvement concerns various psychological factors relevant to demonstrative choice. We show that [+distal] demonstratives, like [+distal] complementizers in the sentential domain, tend to be used more when the Addressee is more involved in the conversation. In Section 6 we return to the matrix in Table 1 to explain some gaps. In particular we answer the question why actual distance is not used with anaphoric reference and why *this* cannot be used as a complementizer. We also give a definition of Addressee involvement that derives its interpretation in all four contexts in Table 1. Finally, this section discusses some related work and some final remarks.

## 2 Direct and indirect speech

As mentioned in the introduction, English allows direct speech complements to be introduced by the proximal demonstrative *this*, but not the distal demonstrative *that*. The latter has grammaticalized into a complementizer which can be used to introduce indirect speech, where *this* is not allowed:

(1)

**Table:** 

Sue said (*this/that) it is raining.
(cf. Rooryck 2019: 257)

(2)

**Table:** 

*Sue said (this/#that): “It is raining.”*
(cf. Rooryck 2019: 257)

We argue that this pattern is not arbitrary, but is based on the recycling of the category of physical distance ([±distal]) in grammar. In the case of the distinction between direct and indirect speech, there are two target categories for the recycling process: actual distance and Addressee involvement. Both provide a link between physical distance and the direct/indirect speech distinction.

We begin our discussion with actual distance, which is the most intuitive category in this context. Observe that direct and indirect speech reports differ in the degree to which the report is similar to the original utterance. Indirect speech reports do not need to be very similar to the original utterance; they only need to match their at-issue entailments, implicatures, and presuppositions (Brasoveanu and Farkas 2007). Thus (1) may for example be uttered after Sue has said something like *Why is it always raining when I want to go out?*; this original utterance matches in terms of entailments, implicatures, and presuppositions with the report in (1). However, it cannot be reported with the direct speech report in (2). For a speaker to faithfully utter (2), Sue’s utterance must have been (almost) lexically identical to *It is raining*. It thus becomes clear that direct speech reports do not only have restrictions on the semantic and pragmatic content of the original utterance, but that they have additional constraints on its surface form. In this way, a direct speech report is more similar to the reported utterance than an indirect speech report. As a result, direct speech reports also lend themselves better to “personal” renderings of the original utterance, including the imitation of accents, pitch, accompanying gestures, etc. (Clark and Gerrig 1990). In this way direct speech again allows for greater similarity to the original utterance than indirect speech.

We think of this similarity in the following way. Both the original utterance and the speech report can be defined in terms of properties referring to their precise lexical form, propositional content, entailments, phonological information needed to represent accents, accompanying gestures, and possibly more features. This view of speech reports and utterances as multidimensional objects allows us to compare two of them and evaluate their similarity. This is analogous to defining a point in the physical world with *x*, *y*, and *z* coordinates and measuring the distance between two points.4This is similar to Paul Churchland’s notion of state space (also similarity space). Churchland proposes that “the brain represents various aspects of reality by a *position* in a suitable *state space*” (1986: 280; emphasis original). For example, a color can be defined as a point in a three-dimensional state space, where each dimension measures the degree to which one receptor type is activated. Colors can then be compared as similar or dissimilar by measuring the distance between them in this color space. Churchland proposes state spaces for different sensory systems. He also suggests that concepts can be represented in a state space for language use and propositional knowledge (1986: 299–306), which is what we attempt to do here. The difference is that utterances are represented in a multidimensional conceptual space rather than in a three-dimensional physical world. Nevertheless, this analogy shows that the similarity of a speech report to the original utterance can be seen as the recycling of the actual distance between the referent (the original utterance) and the deictic expression (*this* or *that* in the context of the speech report).5Throughout, we use the term referent for the thing to which the deictic expression refers (cf. Maes et al. 2022). Note that this is different from antecedent, since the referent is not normally a linguistic element but an entity in the speech situation (the physical book with *that book there*) or an utterance or proposition (as with speech reports). We will use the term actual distance to refer to the Euclidean distance both in the physical world and in the multidimensional conceptual space where the similarity of speech reports is assessed in terms of distance to the original. Note that it is also very common to talk about similarity in phonological or propositional form in terms of distance: *You think that’s what he talks like? That doesn’t even come close!* or *You couldn’t be further from the truth*.

The second way in which the proximal/distal distinction is recycled is as Addressee involvement – and this category can be generalized to all other contexts that we discuss in the present article. Addressee involvement is an interpretation of the “distance” between the referent (Sue’s utterance) and the Speaker (of [1]–[2]). A direct speech report as in (2) is “close” to the Speaker, because its use suggests that the Speaker has direct, reliable knowledge of Sue’s utterance. By the Speaker’s uttering of (2), the Addressee also receives evidence for Sue’s utterance, but it is only indirect evidence. The proximity expressed by *this* positions Sue’s utterance close to the Speaker, and reflects that the Speaker has more direct evidence than the Addressee for Sue’s utterance. The Addressee is much less involved. On the other hand, an indirect speech report as in (1) does not imply that the Speaker has direct evidence for the utterance. Speaker and Addressee can then share the indirect evidence: the evidence is in the Shared Discourse Space. Distal *that* positions the complement clause close to the Addressee because the Speaker and the Addressee have the same amount of evidence for the information in that clause, and the Addressee is more involved. Closeness to the Addressee is represented as distance from the Speaker, hence a [+distal] element is used.

**Figure 1:**
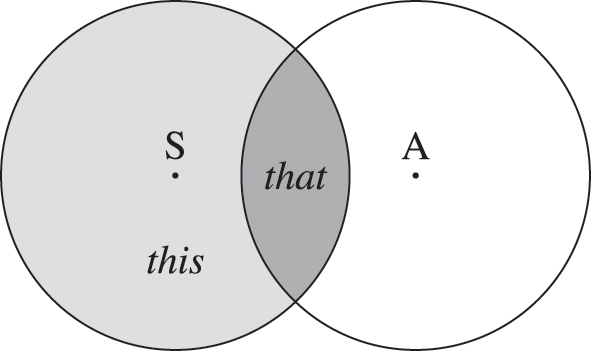
The Personal Discourse Spaces of the Speaker and the Addressee, with the Shared Discourse Space as their intersection.

This view entails, perhaps counter-intuitively, that the Shared Discourse Space is distal for the Speaker. We see the Shared Discourse Space not as a region encompassing Speaker and Addressee, but as the intersection of their Personal Discourse Spaces: the collections of information content tracked by each of the interlocutors individually (including propositions, utterances, questions, …). This is illustrated in Figure 1. The Shared Discourse Space is therefore not proximal for the Speaker, but the proximal/distal distinction is used to distinguish between the information content private to the Speaker (proximal, light gray in Figure 1) and the information content shared with the Addressee (distal, dark gray in Figure 1). In this way, although the speech report is positioned either close to or far from the Speaker, this is actually used to mark its absence or presence in the Addressee’s Personal Discourse Space, respectively. For this reason we speak of Addressee involvement with a focus on the Addressee rather than the Speaker. In the case of speech reports, this Addressee involvement receives an evidential interpretation, with proximity/distance to the Speaker being recycled for direct/indirect evidentiality. As we shall see below, Addressee involvement receives a different interpretation in other contexts.

By way of definition, it is useful to compare our model of the Shared Discourse Space to current approaches to the Common Ground (e.g. Clark 1996; Farkas and Bruce 2010; Lewis 1969; Stalnaker 1978). First of all, the notion of Shared Discourse Space is broader than that of Common Ground. For Farkas and Bruce (2010) the Common Ground is the intersection of the interlocutors’ commitment sets, and the commitment set of an interlocutor consists of the propositions she has publicly committed to. Because the commitment set is defined in terms of public commitments and not knowledge or belief, conversation participants know the contents of each other’s commitment sets. But the focus on commitment to propositions makes this model too constrained for our purposes. For our analysis of demonstratives in Sections 4 and 5 it will be necessary to also include referents of demonstratives in the Shared Discourse Space. Furthermore, the sensitivity of complementizers to previous questions or utterances explored in Sections 3.2 and 3.3 also requires a notion broader than commitment to propositions. For this reason, the Shared Discourse Space does not (only) contain information about commitment to propositions, but more generally about all the entities and information content that are tracked by the interlocutors as part of the discourse context. The Personal Discourse Space of an interlocutor consists of the entities and information content that are tracked by her – and we take tracking *x* to be general enough to include believing *x*, believing that ¬*x*, being interested in whether *x* is the case, pondering the requirements or corollaries of *x*, having any kind of emotional attitude towards *x*, etc. It therefore includes, but is not limited to, the Common Ground. It is also not limited to propositions: it may contain *x* if the interlocutor tracks that *x* has (or has not) been uttered, that *x* did (did not, might, should, etc.) occur, or how *x* can be identified. The Personal Discourse Space contains the entities and information content to which an interlocutor is, in a broad sense, attentive. The Shared Discourse Space, then, consists of the entities and information content that are tracked by the Speaker and that are assumed (by the Speaker) to be tracked by the Addressee.6Note that this derives the fact that there is no extra-distal demonstrative to refer to something in the Personal Discourse Space of the Addressee but not in that of the Speaker: as soon as something in this region is referred to by the Speaker, it becomes part of her Personal Discourse Space as well, because it becomes tracked. There is no way for the Speaker to talk about something without tracking it herself.


We approach the Shared Discourse Space explicitly from the point of view of the Speaker: the Shared Discourse Space is the intersection of that which the Speaker considers her Personal Discourse Space and that which she assumes is the Addressee’s Personal Discourse Space. Others already recognized the need for the perspective of the Speaker in the analysis of Common Ground. For instance, Clark (1996: 96) notes that only an omniscient being can say “It is common ground for the two of them that […]”, and conversation participants can only say “I believe that it is common ground for us that […]”. Clark recognizes that there may be situations where the interlocutors have different ideas of what the Common Ground contains. In such situations, the language used by the interlocutors is determined by the assumptions they make about the Common Ground – not by what an omniscient being would theoretically know that the Common Ground consists of. The same applies by extension to the Shared Discourse Space. Stalnaker (1978: 321) seems to recognize the same thing when he writes that “presuppositions are what is *taken by the speaker* to be the common ground” (emphasis ours), but later defines Common Ground without taking into account the perspective of the conversation participants: “the common beliefs of the parties to a conversation are the beliefs they share, and that they recognize they share” (Stalnaker 2002: 704). This type of Common Ground only exists as a theoretical construct, it is inaccessible to the interlocutors and therefore cannot influence the way they speak. For this reason, we explicitly take the Speaker’s perspective on the Shared Discourse Space.

The treatment of speech reports and demonstratives proposed in this section has many precursors in the literature. For instance, Clark and Gerrig (1990: 792–793) observed that the Speaker of a direct speech report takes responsibility for the correct rendering of an utterance, while the Speaker of an indirect speech report takes responsibility for the interpretation of an utterance. Wierzbicka (1988: 132–135) has an analysis of indirect speech *that* which is similar to ours, although she compares it to direct speech introduced with a pause rather than proximal *this*. She argues that direct speech reports “sound like reports of utterances expressing emotion, rather than ‘objective’ judgement”, while indirect speech reports “imply that the speaker was trying to assess the reality, not merely to express his emotion” (1988: 133). For instance, utterances that are high in emotive attitude, like *You idiot!*, can hardly be reported with indirect speech (?*He said that she was an idiot*; preferred would be: *He called her an idiot*). This can be seen as a reluctance to refer to the meaning of emotive utterances as opposed to the utterance itself. This reluctance would be understandable: if the original Speaker made an emotive utterance, she may not be held fully responsible for its propositional content because the utterance may be made in the heat of the moment. However, neither Clark and Gerrig (1990) nor Wierzbicka (1988) related these observations to the proximal/distal distinction that remains present in complementizers. Rooryck (2019: 256–257) does discuss speech reports with reference to the proximal/distal distinction, but considered the Common Ground to be proximal to the Speaker.7In Rooryck (2019: 256) it was suggested that proximal *this* places the content of a direct speech report in the Common Ground, because the Common Ground is proximal to Speaker and Addressee. By contrast, we take *that* to involve reference to the Shared Discourse Space while *this* refers to the Personal Discourse Space, i.e., to information content tracked by the Speaker but not shared by the Addressee. Therefore, for reasons outlined above, direct speech reports are not placed in the Shared Discourse Space but remain personal to the Speaker, while indirect speech reports are shared with the Addressee.


## 3 Presupposition effects

Having shown how the [±distal] feature is recycled to mark actual distance (interpreted as similarity) and Addressee involvement in the context of speech reports, we now turn to cases where overt complementizers contrast with zero complementizers.8Depending on one’s syntactic framework, these cases could also be analyzed as contrasts between an overt complementizer and the lack of a complementizer. This does not affect the argument: in the end it is the presence (or absence) of the [+distal] feature that matters. We will use “zero complementizer” for simplicity, without making any theoretical assumptions. In these cases there is no difference in terms of actual distance, but the notion of Addressee involvement does generalize.9We return to the question why actual distance is not relevant here in the conclusion. Our position will be that overt complementizers which are historically based on non-proximal elements markedly involve the Addressee. In particular, we analyze the examples below using the notion of Shared Discourse Space. When information content is in the Shared Discourse Space, it is shared with the Addressee, and therefore “far” from the Speaker; when information content is not in the Shared Discourse Space but is tracked by the Speaker alone, it is instead “close” to the Speaker. The proximal/distal distinction is thus recycled to indicate the absence/presence of content in the Shared Discourse Space.

### 3.1 Exclamatives

We first look at main clauses with overt complementizers, which in many languages can get an exclamative reading:10However, constructions in Romance of the type *Que cette histoire est obscure!* ‘How dark this story is!’ (French) should be kept separate, because they always refer to a degree rather than a fact (Trotzke and Villalba 2021). We are grateful to Maria Bardají i Farré for suggesting this reference.

(3)

**Table:** 

a.	*That bio industry is still allowed!*
b.	*That he should have left without asking me!*
	(Quirk et al. 1985: 841 via Zevakhina 2013: 167)

(4)

**Table:** 

*Att*	*du*	*hann*	*med*	*tåg-et!*	
comp	you	reach.pst	with	train-def	
‘(It is surprising,) that you caught the train!’					
(Delsing 2010: 17 via Zevakhina 2013: 167)					(Swedish, IE/Germanic)

(5)

**Table:** 

*Że*	*też*	*potrafiłeś*	*coś*	*takiego*	*zrobić!*	
comp	also	can.pst.2m.sg	something	this	do.inf	
‘That you could do something like this!’						
(based on Storms 1966: 261^11^)						(Polish, IE/Slavic)

11 We are grateful to Justyna Visscher-Jablonska for providing a Modern Polish version and glosses.

(6)

**Table:** 

*zaʕaqa-ṯ*	*sәḏōm*	*wa=ʕămōrͻ*	*kı̄*	*rͻbb-ͻ*	*wә=ḥaṭṭͻṯ-ͻm*	
outcry-of	Sodom	and=Gomorrah	comp	great-3f.sg	and=sin-3m.pl.poss	

*kı̄*	*ḵͻḇәḏ-ͻ*	*mәʔōḏ*				
comp	heavy-3f.sg	very				
‘The outcry of (/against) Sodom and Gomorrah, how great it is! And their sin, how very grievous!’							
(Genesis 18:20)						(Biblical Hebrew, Semitic)

In these examples, the exclamative is only distinguished from a regular declarative sentence by the addition of the complementizer and a different intonation pattern. The intonation pattern alone is not enough for the exclamative interpretation. For instance, a sentence like *Bio industry is still allowed!*, with the same intonation pattern as the exclamative, still differs from an actual exclamative like (3a) in that it can be used to attempt to convince the Addressee of its propositional content. By contrast, the sentence in (3a) does not make an attempt at informing or convincing the Addressee of its propositional content, but actually presupposes it to be a shared presupposition in the Common Ground, and hence in the Shared Discourse Space. The use of the complementizer is therefore crucial for the interpretation as an exclamative. Zanuttini and Portner (2003) already showed that exclamatives are factive.12In terms of Ross’s (1970) performative hypothesis, factivity of exclamatives would be explained through the deletion of a factive performative (*I am surprised that …!* > *that …!*); see also Evans (2007) for diachronic considerations. In neo-performative treatments the performative structure is not deleted but part of the functional domain above the CP (e.g. Speas and Tenny 2003). Alternatively, *that* could be seen as an underspecified element, with its factive meaning deriving from the merge site (cf. Kocher 2022 on Ibero-Romance *que*). The exact derivation of exclamatives is not relevant here; what is important is primarily the fact that exclamatives are factive. On this view, exclamatives make reference to a proposition that is already presupposed in the Shared Discourse Space and relate a certain Speaker stance (surprise, anger, etc.) to it.13In some cases, the proposition is strictly speaking not presupposed but can be easily accommodated by all interlocutors. We see such cases as involving an imposition on the Common Ground, and by extension on the Shared Discourse Space, through referencing a proposition: by referencing the proposition, the Speaker pretends that it is already in the Common Ground, thereby imposing an update to the Common Ground. Also see our discussion of Kocher (2022) in Section 3.3 below, especially footnote ^25^. We propose that the [+distal] complementizer in these exclamatives anaphorically refers to the presupposed proposition.14Note that other syntactic strategies of exclamatives studied by Zevakhina (2013) also contain other anaphoric elements:

(i)

**Table:** 

*Zhège*	*háizi!*	
this	child	

‘What a child!’					
(Visan 2000: 9 via Zevakhina 2013: 169)					(Mandarin Chinese, Sino-Tibetan)

(ii)

**Table:** 

*It’s so hot!*
(Michaelis 2001: 1040 via Zevakhina 2013: 166)

(iii)

**Table:** 

*Miša*	*takoj*	*bol’šoj!*	
Miša	such.nom	big.nom	

‘Miša is so big!’													
(Zevakhina 2013: 166)													(Russian, IE/Slavic)

Although these anaphoric elements do not refer to a presupposed proposition, they still establish Shared Discourse Space between Speaker and Addressee.


In these cases, the referent (the presupposition in the Shared Discourse Space) is always “far” from the Speaker: we do not find exclamatives with a complementizer or other grammatical marker that is specified for [−distal].15See example (i) in footnote ^14^ for a case where a proximal element can be used in an exclamative. But note that this is an exophoric demonstrative and does not head the exclamative clause. The notion of Addressee involvement makes it easy to see why: if, following Zanuttini and Portner (2003), exclamatives require presupposition, they must refer to the Common Ground, and hence to the Shared Discourse Space. An exclamative cannot at the same moment introduce new, Speaker-personal information content into the discourse. As a result, the information content must be close to the Addressee, and therefore a distal element must be used.16Ellen Brandner (p.c., August 26, 2022) notes that in some German exclamatives the distal demonstrative *der* is preferred over the personal pronoun *er* ‘he’, as in *der*/#*er und lesen!* ‘he and reading!’ (i.e., he will definitely not read; the idea is preposterous). The preference for the distal demonstrative cannot be explained by emotional distancing from the subject, as the same effect appears with predicates with a negative connotation: *der*/#*er und Plagiat begehen!* ‘he and committing plagiarism!’ (i.e., he will definitely not commit plagiarism, which is a meliorative statement and would not require emotional distancing). The affinity of exclamatives with distal elements may thus extend beyond the complementizer.


### 3.2 The *that*/zero alternation in English object clauses

We can also use Addressee involvement to explain the alternation between overt and zero complementizers in English object clauses. Consider (7):

(7)

a.

**Table:** 

*I thought you might need some help.*
(Bolinger 1972: 58)

b.

**Table:** 

*I thought that you might need some help.*
(Bolinger 1972: 58)

A common view is that the complementizer *that* in (7b) is “optional”, i.e., that its use is determined by style or register and that it does not have an interpretive value. However, the literature discusses many factors that can play a role in the choice between *that* and a zero complementizer. Two in particular suggest that we are actually dealing with an interpretively meaningful alternation and not with an entirely optional functional element.17We are not concerned here with cases where *that* is used to avoid ambiguity or otherwise make parsing the sentence easier (e.g., Bolinger 1972: 18–42; Elsness 1984). Beal (1988: 60) and Rissanen (1991) observed that *that* is more often omitted in constructions that frequently take complement clauses, because the pattern is less unexpected and does not need to be marked by *that*. We take this to indicate that *that* is inserted in infrequent collocations to clarify the sentence structure (cf. also Kajzer-Wietrzny 2018). Although these factors are not relevant to us here, one should be aware of their existence because they can interfere with minimal pairs. We also set aside here style and register (Elsness 1984; Rissanen 1991), as well as the suggestion found in Hooper and Thompson (1973), Thompson and Mulac (1991), Diessel and Tomasello (2001), and Thompson (2002) that certain combinations of first and second person subjects and verbs like *think* and *guess* can be reanalyzed as markers of epistemic modality so that the distinction between main and complement clause erodes and *that* is less likely (Kaltenböck 2009 and Dehé and Wichmann 2010 seek to predict when clause-initial constructions like *I think* (*that*) are such epistemic markers, as opposed to matrix clauses).


Firstly, Bolinger (1972: 58) already noticed that the sentence with *that* in (7b) suggests some context between Speaker and Addressee. This context may be extralinguistic, as in the scenario he sketches:18
Bolinger (1972) contains many more examples. Some native speakers we consulted do not share Bolinger’s intuition expressed here. This may be due to the fact that there are many different factors that play a role in the choice between *that* and zero. The relative weight of these factors could vary between speakers and variants of English. More work is needed to establish the extent to which Bolinger’s intuitions are still relevant today. For the present study, it suffices to say that shared context between Speaker and Addressee played a role in at least one variety of English at one point in time. “Suppose you observe a stranger struggling to mount a tire. Feeling charitable you go over to him and say [7a]. Under these circumstances, [7b] would be inappropriate. But if the other person looks at you as if wondering why you came over, you might explain by saying [7b]” (Bolinger 1972: 58, example numbers adapted).

In the words of Bolinger (1972: 56), the complementizer still “reflects the demonstrative character of *that*” in that it refers to this shared context. After all, this use of *that* appears to be quite similar to the discourse deictic function of demonstratives (e.g. *That’s a lie*; Diessel 1999: 101). Both refer to some utterance, even though the utterance is only implied in (7b) (i.e., we assume there to be an implicit utterance along the lines of *Why did you come over?*). The situation is then quite similar to that of exclamatives: the use of an overt complementizer signals content in the Shared Discourse Space. Again, then, the referent (the presupposed utterance) is analyzed as “far” from the Speaker, triggering a distal element, because it is in the Shared Discourse Space, close to the Addressee. In (7a), no anaphoric element is present because the idea that the Addressee might need help has not yet been introduced, and is therefore not in the Shared Discourse Space. The presence or absence of the complementizer thus marks the presence or absence of shared context in the Shared Discourse Space.19According to Auer (1998), cited by Weinert (2012), something similar is the case with German *dass* ‘that’: “unintroduced main clauses are relatively assertional (they tend to contain fore-grounded and new information) whereas introduced complement clauses are relatively presuppositional (they tend to contain back-grounded and known information)” (Weinert 2012: 243).


The other relevant factor conditioning the choice between *that* and zero is that of subjectivity (Storms 1966: 262–265). Storms argues that sentences incorporating a *that*-clause are “less personal, less familiar, less warm, less friendly, less emotive” than their counterparts with zero complementizers (Storms 1966: 262). He gives examples from a witness interrogation in court, where sentences without *that* are used “to put the witness at her ease and at the same time to set an unsuspected trap” (Storms 1966: 263). Later, when it is important that objective facts are established, questions with *that* are used (Storms 1966: 264). Similar ideas appear in Wierzbicka (1988: 132–140), who relates *that*-clauses (as opposed to other complementation types) to knowledge. We believe that this subjectivity derives from the placement of the complement in or outside of the Shared Discourse Space. The lawyer cited by Storms (1966) uses *that* for propositions that are not yet in the Common Ground, but by using *that* he implicitly proposes to update the Common Ground to include them.20The use of *that* in “less friendly” contexts could also be related to the formal register with which *that* is associated. However, conversely it may also be the case that *that* is associated with formal language precisely because of this interaction with “subjectivity”.


Previously, Kaltenböck (2006) already proposed to use the abstract notion of distance to explain the difference between *that* and zero in extraposed *that*-clauses (as in *It is obvious* (*that*) *she did it*), suggesting that the analysis could be generalized to object clauses as well (Kaltenböck 2006: 389 n. 20). In his view, the abstract notion of distance is interpreted as one or more of (a) illocutionary distance (asserting the complement with zero vs. disposing the matrix for illocutionary force with *that*), (b) temporal/anaphoric distance (using *that* for complement clauses whose content has already been talked about vs. zero for new information), and (c) emotional distance (à la Storms 1966). However, Kaltenböck (2006) does not provide a principled reason why old information should be distal (in terms of temporal/anaphoric distance); we might as well argue that discourse-old information is proximal, because what is close to us is better known than what is far. The notion of Addressee involvement provides an explanation: discourse-old information is distal because it is in the Shared Discourse Space, known and shared by the Addressee. Addressee involvement is also needed to explain the lack of a proximal complementizer *this*, which Kaltenböck’s (2006) analysis does not seem to predict. We return to this issue in Section 3.4. We discuss more related work in Section 6.2.

To finish our discussion of optional *that* in object clauses we briefly discuss the fact that *that* is required when the object clause is topicalized:

(8)

**Table:** 

a.	*I always believed (that) the jury was bribed.*
b.	*(That) the jury was bribed, I always believed. ^21^

21 *That*-deletion is actually obligatory in this example if *I always believed* is taken as an evidential modifier (cf. *The jury was bribed, I think*). However, in such a case *that* is not permissible because *The jury was bribed* is the main clause (cf. Bolinger 1972: 15–16, 62; Hooper and Thompson 1973; Thompson and Mulac 1991). The two can be distinguished by the fact that the sentence with an object clause does not make an attempt to update the Common Ground: (8a) is acceptable in contexts where the Addressee does not need to accept that the jury was bribed. For instance, *whatever* in *I always believed the jury was bribed, but whatever* indicates that the Speaker does not care about the Addressee’s commitment to the proposition that the jury was bribed, whereas *whatever* in *The jury was bribed, I always believed, but whatever* indicates that the Speaker does not care about the fact that the jury was bribed. We are concerned here with the sentence with topicalization, which does make an attempt to update the Common Ground and in which *that*-deletion is optional.

As discussed by Rizzi in Kratzer et al. (2020), there have been different syntactic accounts of this phenomenon. In this interview, Rizzi proposes an account that adopts an idea from Pesetsky (1994). Rizzi assumes that the lack of a complementizer indicates incorporation or cliticization of that complementizer into the selecting verb. Since the C head of the complement clause has already moved, the complementizerless clause cannot in turn move to a higher position: the complement clause is frozen in place. This would explain the pattern in (8). However, this account needs to introduce the otherwise uncorroborated assumption that complementizers incorporate into the selecting verb in English. By contrast, the analysis based on Shared Discourse Space that we present here suggests an explanation that derives from a wider generalization: *that* is required in topicalized object clauses because topicalizations are necessarily discourse-old, and hence in the Shared Discourse Space. The complementizer *that* is then required to indicate the shared status of the topicalized clause.

### 3.3 Overt root complementizers in Romance

Finally, presupposition and the Shared Discourse Space also play a role in constructions with a root complementizer found in several Romance languages. It may not be immediately obvious that the complementizers discussed here contain a non-proximal deictic element, but we return to this issue below. One type of root complementizer construction that we are interested in here has a sentence-initial adverb followed by an overt complementizer:22Other, similar constructions are discussed in depth by Kocher (2022: 91–196). She shows that all these cases impose a commitment to a proposition on the part of the Addressee, which is a clear case of Addressee involvement and very similar to the cases discussed above. For reasons of space we discuss only one construction here.

(9)

**Table:** 

*Evidentemente*	*(que)*	*Julia*	*está*	*muy*	*enfadada.*	
obviously	comp	Julia	is	very	angry	
‘Obviously, Julia is very angry.’						
(Etxepare 1997: 98–99 via Hernanz 2007: 165–166)						(Spanish, IE/Romance)

According to Etxepare (1997: 99 via Hernanz 2007: 166), the felicity of Spanish *que* in sentences like (9) is conditioned by the occurrence of a clear “linguistic antecedent” in the preceding discourse (cf. Etxepare 2010: 613). Thus, (9) is only felicitous after another Speaker has uttered a sentence like *¿Se ha enfadado Julia?* ‘Did Julia get angry?’. We might analyze this linguistic antecedent as establishing a Question Under Discussion (QUD) in the Shared Discourse Space.23This treatment is similar to that of Pérez and Verdecchia (2022) for “clausal doubling”, which covers cases like: *Que leyó el libro, seguro que lo leyó* ‘As for her reading the book, she read it for sure’. The first clause is seen as establishing the QUD; the second clause has *que* because it responds to this QUD. The existence of this QUD then licenses the use of *que*. Thus, as in the cases discussed above, the use of an overt complementizer is licensed by the existence of an element in the Shared Discourse Space (namely, the QUD). Because we want to focus here on the properties of the Shared Discourse Space, we do not go into details about a possible formal representation of the QUD.24See e.g. Ginzburg (1996), Roberts (2012 [1996]), Büring (2003), and Farkas and Bruce (2010). What is relevant to us is only that the Shared Discourse Space (a) contains informational elements, (b) that these elements can be tracked and referred to by conversation participants, and (c) that some of these elements can be marked as being under discussion.

According to Kocher (2022: 75–82), the linguistic antecedent requirement is not as strict as assumed by Etxepare (1997, 2010, as *que* can also be used for future or hypothetical utterances:

(10)

**Table:** 

*Avisa*	*el*	*comissari.*	*Que*	*ja*	*pot*	*venir.*	
notify.2sg.imp	the	inspector	comp	already	can.3sg.prs	come	
‘Notify the inspector. [reportative:] He can already come.’							
(Kocher 2022: 77)					(Catalan, IE/Romance)		

In Kocher’s (2022) analysis, *que* merges in a high position in the left periphery in cases like (10), where it simply indicates that the sentence is subordinate. The Addressee can then infer that a *verbum dicendi* is implicitly understood. This is in contrast to cases like (9), where *que* merges in a low position in the left periphery where it expresses that a commitment to the proposition is attributed to the Addressee (an attributive feature in the sense of Poschmann 2008). This attributive feature, which is a form of Addressee involvement, explains why B’s response is felicitous in (11a) below but not (11b). In (11b), the attributive feature of *que* clashes with A’s sentence in which the proposition is described as a false belief:

(11)

a.

**Table:** 

A:	*Qué*	*dicen*	*los*	*doctorandos*	*al*	*inicio*	*de*	*sus*	
	what	say.3pl.prs	the	PhD_students	at_the	beginning	of	their	

	*estudios?*								
	studies								
	‘What do PhD students say at the beginning of their studies?’								

B:	*Que*	*{seguro*	*que*	*/ seguramente}*	*acabarán*	*su*			
	that	sure	comp	surely	finish.fut.3pl.fut	their			

	*tesis*	*a*	*tiempo.*						
	thesis	on	time						
	‘That surely they will finish their thesis on time.’								
(Kocher 2022: 176)									(Spanish, IE/Romance)

b.

**Table:** 

A:	*Cual*	*es*	*la*	*falsa*	*idea*	*que*	*tienen*	*los*	
	what	be.3sg.prs	the	false	idea	that	have.3pl.prs	the	

	*doctorandos*	*al*	*inicio*	*de*	*sus*	*estudios?*			
	PhD_students	at_the	beginning	of	their	studies			
	‘What is the false belief that PhD students have at the beginning of their studies?’								

B:	*Que*	*{#seguro*	*que*	*/ seguramente}*	*acabarán*	*su*			
	that	sure	comp	surely	finish.3pl.fut	their			

	*tesis*	*a*	*tiempo*.						
	thesis	on	time						
	‘That surely (#*que*) they will finish their thesis on time.’								
(Kocher 2022: 175)									(Spanish, IE/Romance)

We are agnostic towards the exact syntactic derivation leading to the attested surface structures. What is important to us here is that in both cases distinguished by Kocher (2022), the use of *que* is conditioned by the existence of shared context between Speaker and Addressee. In cases like (9), the proposition itself is placed in the Shared Discourse Space; in cases like (10), there is the salient *verbum dicendi* that provides the shared context.25A small difference between our account and that of Kocher (2022) is that she describes the pragmatics of attributive *que* as “imposing” a proposition on the Common Ground, whereas we talk about referencing a proposition in the Common Ground (and by extension in the Shared Discourse Space). The term “reference” could seem to suggest that the proposition must already be in the Common Ground. This is, of course, not the case: it is perfectly possible to introduce new information in *que*-clauses. We suggest that this information is introduced by referencing it. The speaker “imposes” it on the Common Ground by pretending that it is already there. We hold on to the term “reference” to highlight the parallels with demonstratives, discussed below. The difference with “imposition” is largely terminological.


We find similar constructions in other Romance languages:26We use an example from Romanian here; for examples from other languages see Cruschina and Remberger (2017). For more discussion on Romanian, see Hill (2012).

(12)

**Table:** 

*Sigur*	*(că)*	*va*	*veni.*					
sure	comp	will.3sg	come					
‘Of course s/he’s coming.’								
(Cruschina and Remberger 2017: 89)								(Romanian, IE/Romance)

In (12), Romanian *că* may only be used when the Addressee could have inferred the propositional content of the clause. Cruschina and Remberger (2017: 89) set up the following contexts. Suppose Ioana asks Alexandru if Ion will attend a conference next week. Ioana does not and cannot have this information, but Alexandru has spoken to Ion and knows that he is coming. Alexandru can then answer with *Sigur va veni*. However, suppose now that Alexandru does not have this information, but that both Ioana and Alexandru know that Ion is a big fan of the conference and would never miss it. In this context, Alexandru can answer with *Sigur că va veni*. The answer is then marked as an inference from information in the Common Ground between Ioana and Alexandru, rather than as private information of Alexandru. Again, we see that reference to the Shared Discourse Space (here in particular the Common Ground), and hence Addressee involvement, is marked by an overt complementizer.

A related phenomenon can be observed in Neapolitan. The following contrast is discussed by Sornicola (1996: 334–336) and Ledgeway (2011: 286–289):

(13)

a.

**Table:** 

*Chillo*_i_	*s’è*	*astutato*	[*’o*	*riscaldamento*]_i_	
that.m	self=is	turned_off	the.m.sg	heating.sg	
‘The heating has gone off.’					
(Ledgeway 2011: 286)					(Neapolitan, IE/Romance)

b.

**Table:** 

*Chello*_i_	*s’è*	*astutato*	[*’o*	*riscaldamento*]_j_	
that.n	self=is	turned_off	the.m.sg	heating.sg	
‘(The fact is/Because) the heating has gone off.’					
(Ledgeway 2011: 286^27^)					(Neapolitan, IE/Romance)

27 The indices in (13b) have been corrected from the source after consultation with Adam Ledgeway (p.c., June 16, 2022).

On Ledgeway’s double subject analysis, *chello*/*chillo* is not a complementizer but a demonstrative, but it is still similar to the cases discussed above. In (13a), *chillo* is coreferential with the second subject (‘It has gone off, the heating’). In (13b), neuter *chello* cannot be coreferential with masculine *’o riscaldamento*. The demonstrative must therefore refer to something else. It has “a distinctly explicative or adversative value, only proving felicitous in contexts that contain an implicit or explicit presupposition” (Ledgeway 2011: 287). We suggest that in (13b) the demonstrative refers to this associated presupposition, as is the case with the complementizers in Spanish, Catalan, and Romanian.

We have largely left French aside in the discussion above, because French *que* is nearly obligatory in all environments. There appear to be some varieties that do allow *que* to be dropped in some contexts.28Tabea Ihsane (p.c., August 25, 2022) kindly shared an observation that in some modern varieties of French it does seem to be possible to drop *que* under certain circumstances. We also thank Alina McLellan for discussing the situation in Réunion Creole, where *ke* (<Fr. *que*) is optional in many contexts (cf. Corne 1995). However, these cases have not been described in sufficient detail yet to be included in our discussion here.

We should pause here for a moment to reflect on the origin of these complementizers. Above, we argued that the [+distal] complementizer *that* marks the use of Shared Discourse Space because the Shared Discourse Space includes the Addressee and is therefore “far” from the Speaker. However, the Romance complementizers here are not demonstrative synchronically, so how does a [±distal] feature fit in? Note that these complementizers derive from Latin *quod*, which is composed of an interrogative element *qu-* and the originally neuter medial demonstrative *id*. Given the latter component, these complementizers do diachronically derive from a non-proximal demonstrative element. Furthermore, there is reason to believe that the interrogative element *qu-* is incompatible with proximity. For instance, consider that English has *what* from *that* and *where* from *there*, but not *whis from *this* or *where with an /i/-vowel from *here*. Rooryck (2003: 11–12) suggests that this is because something that is proximate to the Speaker is necessarily known to them. In this way the interrogative element *qu-* could also be seen as a [+distal] component, thus involving the Addressee.

Our analysis of these complementizers is very similar to that of exclamatives. In (12), the sentence without *că* has an “objective” interpretation (“It is certain that s/he’s coming”), whereas *că* triggers a “subjective, speaker-oriented” interpretation (“Of course s/he’s coming”), where the propositional content is inferred (Cruschina and Remberger 2017: 88–89). This Speaker-oriented interpretation uses *că* to refer to a presupposition, just as exclamative complementizers refer to the proposition presupposed by their complement. This is entirely in line with Gutiérrez-Rexach (2001: 184–186), who calls these sentences in Romance “evidential exclamatives” and analyzes them as in (14a):

(14)

a.

**Table:** 

[_Force_ Adv/A_[+evident]_ [_Focus_ [+f] [_Topic_ COMP …]]]

b.

**Table:** 

*¡Claro*	*que*	*te*	*lo*	*voy*	*a*	*dar!*	
clear	that	to_you	it	go	to	give	
‘Of course I will give it to you!’							
(Gutiérrez-Rexach 2001: 184–185)							(Spanish, IE/Romance)

According to Gutíerrez-Rexach, the evidential adverb *claro* ‘clearly, of course’ requires that its complement makes reference to some Question Under Discussion (QUD). For example, (14b) may be uttered if the Speaker has borrowed something from the Addressee and the Addressee has expressed doubts about getting it back. The QUD is topicalized by the complementizer *que*. Because the complementizer is demonstrative, the QUD does not need to be spelled out, but the complementizer does need to be overt. The complementizer effectively points to the QUD in the Shared Discourse Space. Note, however, that the fact that it points to the QUD (and not any other element of the Shared Discourse Space) appears to be a language-particular constraint: it applies in Spanish, Catalan, and Neapolitan, but not in Romanian, where *că* does not refer to a QUD but to any evidential basis for the claim made in the complement clause. What is at issue for us here is the generalization that the complementizer points to an element of the Shared Discourse Space.

Example (14b) illustrates the division of labor between the sentence-initial adverb and the complementizer, as well as the parallel with the exclamatives discussed in Section 3.1. As with exclamatives, the function of the complementizer is to mark the existence of Shared Discourse Space between Speaker and Addressee. The sentence-initial adverb in the Romance constructions only specifies the evidential interpretation.29Note that Cruschina and Remberger’s (2017) term “Speaker-oriented” for these evidential sentences refers to the fact that the Speaker makes an inference on the basis of the presupposed proposition. The proposition itself is presupposed, and therefore necessarily not Speaker-oriented, but shared between Speaker and Addressee. This leads to the odd situation that a distal element, which is typically used to trigger a more objective interpretation by placing something in the Shared Discourse Space, actually triggers a “Speaker-oriented” reading. The [+distal] element is therefore again used to signal Addressee involvement.

### 3.4 Presupposition effects: summary

To summarize the findings from this section: evidence from a variety of constructions (exclamatives, English “optional” *that*, and the root complementizer constructions in Romance) suggests that the alternation between an overt complementizer with a [+distal] feature and a zero complementizer is related to presupposition. We explain this by suggesting that the complementizer refers to information content in the Shared Discourse Space. Distal elements are used in these complementizers because the Shared Discourse Space includes the Addressee, who is “far” from the Speaker. Note that the theory correctly predicts that we do not find [−distal] elements in these environments, that is, that there is no complementizer derived from the demonstrative *this*. These would correspond to presuppositions that are not shared with the Addressee; a contradiction in terms, since presuppositions are necessarily assumed to be shared by all interlocutors. The only available alternation is with a zero complementizer, which marks the absence of a presupposition from both the Shared Discourse Space and the Personal Discourse Space.

Our analysis raises questions for the traditional account of the grammaticalization of *that* and cognate complementizers. The traditional view is that *that* became a complementizer as a result of reanalysis of a cataphoric demonstrative: *I say that: he comes* > *I say that he comes* (e.g. Diessel 1999: 123–125; Roberts and Roussou 2003: 113–120). As a cataphoric demonstrative, *that* introduces new information, which would be consistent with the use in *I say that: he comes*. But the shift to *I say that he comes* would be odd if the complementizer *that*, as in our analysis, refers to Shared Discourse Space (as opposed to introducing new information).30Another issue with this diachronic account is that *I say that: he comes* is less natural than *I say this: he comes*, while *this* did not grammaticalize into a complementizer (Kayne 2014: 189). However, recent studies have suggested that the complementizer *that* instead developed from a correlative construction: *I say that, that he comes* (e.g. Axel-Tober 2017, and see Bate In preparation for a survey of finite complementizers in Indo-European). In such a construction, the first pronoun introduces new information but the second can be seen as referring to the Shared Discourse Space (as established by the first pronoun). This grammaticalization path therefore does not suffer from the same problem. Our analysis provides further support for this development.

Finally, although we focus here on complementizers derived from demonstrative pronouns, the phenomenon that finite complementizers are related to presupposition seems to be more general than that. For example, the Bulgarian relativizer *deto* (lit. ‘where the’, i.e. ‘the place where’) is also used to express Speaker stance about presupposed propositions: *Săžaljavam, deto ne možax da dojda* ‘I regret that I couldn’t come’ (Krapova 2010: 1240). It may therefore be that a finite complementizer does not need to be derived from a demonstrative pronoun, but that any deictic origin would suffice. We will not explore this further here.

## 4 Exophoric demonstratives

In Sections 2 and 3 we examined the complementizer *that*. We compared this functional element to both the proximal cataphoric demonstrative *this* (for direct speech) and a zero complementizer (in main and object clauses). Both sections were concerned with reference to information content, namely, the meaning of utterances (which may or may not be in the Shared Discourse Space). We now move on to discuss reference to entities in the speech situation. In this context, we are concerned with the demonstrative *that* (and *this*) rather than the complementizer. Here, too, we make a distinction between two types of reference: exophoric demonstratives referring directly to entities in the speech situation (discussed in this section) and anaphoric demonstratives referring to entities as represented in surrounding discourse (discussed in Section 5).

Demonstratives are exophoric when they refer to entities “in the speech situation” (Diessel 1999: 93).31It should be noted that anaphoric demonstratives could also be said to refer to entities in the speech situation, but only indirectly, via an antecedent in the surrounding discourse. This is the prototypical use of demonstratives (e.g. *this*/*that book*) and can be accompanied by a pointing gesture. Traditionally, the distinction between the exophoric demonstratives *this* and *that* is taken to indicate the physical distance between the referent and the deictic origo (typically, the Speaker). However, a wealth of experimental studies have shown this view to be too simplistic (Peeters et al. 2021). Physical aspects of the relation between Speaker and referent are only one of a number of factors determining the choice of demonstrative. There are also psychological factors at play, which relate to “*the cognitive status of the referent* in the mind of the speaker and/or the addressee as assumed by the speaker” (Peeters et al. 2021: 412, emphasis original).32The choice of a demonstrative also depends on referent-intrinsic factors like animacy and grammatical gender, but these are not relevant to us here. For example, different demonstratives may be chosen depending on whether the referent is in joint attention or whether it is considered cognitively accessible by the Addressee (Peeters et al. 2021: 413 and references therein).

Depending on context, different factors may weigh more or less heavily in the choice for a particular demonstrative. Peeters et al. (2021: 416–419) show how this works in Spanish, a language with a three-term distance contrast between *este* (proximal), *ese* (medial), and *aquel* (distal). In an experimental setting where a Speaker has to indicate one of a number of objects to an Addressee across the table, Coventry et al. (2008) found that *este* can only be used for objects in a relatively small zone around the Speaker, excluding most of the table and the Addressee on the other side. At first sight, this seems to be at odds with Jungbluth (2003), who showed that the range of *este* encompasses the entire conversational dyad, including both Speaker and Addressee. However, unlike Coventry et al. (2008), Jungbluth (2003) relies on natural data. Peeters et al. (2021) argue that psychological factors are not available in Coventry et al.’s (2008) experimental setting, prompting interlocutors to interpret the proximal/medial/distal distinction using physical factors like distance, and “calibrating” the different demonstratives to maximize information density. In natural language, however, psychological factors are more important, which explains the different results found by Jungbluth (2003).

In our analysis, psychological factors correspond to Addressee involvement, i.e. the recycling of the spatial relation between referent and Speaker to indicate whether the referent is “shared” with the Addressee. Entities are psychologically further from an interlocutor when they are not in attention or less accessible or identifiable. As above, we propose that English *that* refers to an element of the Shared Discourse Space, while *this* refers to an element in the Speaker’s Personal Discourse Space. These psychological factors can be further interpreted pragmatically. Consider the following examples:

(15)

a.

**Table:** 

*How’s that throat?*
(Lakoff 1974 via Cheshire 1996: 376)

b.

**Table:** 

*How is that term paper coming along?*
(E. Riddle, p.c., via Chen 1990: 150)

The demonstrative in (15a) could in principle be replaced by *your* or *the*. According to Cheshire (1996: 376), *your* would be unmarked, simply indicating awareness of the Addressee’s illness, while *the* would make previous knowledge of the illness explicit. According to her analysis, *that* not only signals this previous knowledge but also expresses Speaker involvement which can be interpreted as empathy with the Addressee. Example (15b) can be analyzed analogously. Using our terminology, we could say that the Speaker uses *that* to signal that the throat is in the Addressee’s and their own joint attention, and that this joint attention is what triggers the sympathetic reading. Lakoff (1974) and Cheshire (1996) do not discuss the interpretation of (15a) with *this*. This sentence seems quite unnatural, but we could imagine (*Let’s see,*) *how’s this throat?* in a context where a doctor begins to physically imagine a patient’s throat. In this situation, the doctor is not interested in the patient’s own judgment – and this corresponds to the lack of Addressee involvement marked by [−distal] *this*.

Kirsner (1979) discusses examples such as the following in Dutch:

(16)

a.

**Table:** 

*Het is smoorheet, iedereen puft en bakt en in die/?deze hitte moet ik alles belopen.*	
‘It is boiling hot, we are all positively melting, and in *that*/?*this* heat I have to walk everywhere.’	
(Anne Frank, 1959, *Het Achterhuis* [The diary of a young girl], cited by	
Kirsner 1979: 357)	(Dutch, IE/Germanic)

b.

**Table:** 

“Ha die/*deze Frits!” zei de jongen, gaf hem een harde klap op de schouder, bleef voor hem staan en zei …	
“‘Aha, (*that*/*this) Frits!,” the boy said, slapped him on the shoulder, remained standing right in front of him and said …’	
(G. van het Reve, 1961, *De avonden* [The evenings], cited by	
Kirsner 1979: 357)	(Dutch, IE/Germanic)

In neither case can the use of the distal demonstrative be explained using physical distance: in (16a), the heat is immediately experienced by the Speaker, and in (16b), the Speaker must be close to the Addressee (given that he slaps him on the shoulder). Instead, Kirsner (1979) proposes that the proximal demonstrative indicates that the Addressee must do relatively much work to identify the referent, compared to when the distal demonstrative is used. This is consistent with our notion of Addressee involvement: in our view, the referent of a distal demonstrative is already tracked by the Addressee, and would therefore require less work to identify.

A somewhat intuitive explanation for the contrasts in (16) (which we do not support) relies on emotional distancing. Similar to Chen (1990) for other examples we might suggest that the use of a [+distal] demonstrative in (16a) creates distance between the Speaker and the referent, because the Speaker has a negative attitude towards the heat. Note, however, that in (16b) the [+distal] demonstrative is used in an intimate, amicable greeting. Chen (1990) simply suggests that *that* can express both emotional distancing and sympathy. But since these two are near polar opposites, this seems unlikely to us. We do not deny that *that* can be used in both positive and negative contexts, but we reject the analysis in which *that* can express both a positive and a negative attitude. Instead, *that* could express a more general notion, and the specific attitude could be derived from this general notion in conjunction with context. Cheshire (1996: 377) calls this notion “interpersonal involvement”. We see it as an instance of Addressee involvement, since in both cases the Speaker is assuming shared context with the Addressee.

Let us then turn to the physical factors determining the choice of the demonstrative. In our model, these correspond to actual distance, i.e. the recycling of the spatial relation between the deictic expression and its referent. Note that exophoric demonstratives are often if not always accompanied by a pointing gesture, and can even be replaced by one (Jouitteau 2004: 431). We take this as an indication that the demonstrative has a position in the physical world, like the referent. Therefore, actual distance, that is, the relationship between the referent (the entity) and the deictic expression (the demonstrative), is determined by physical factors like Euclidean distance in the real world.33Physical factors also include things like visibility, knownness, and elevation (Diessel 1999: 35–47; Peeters et al. 2021), but we focus on physical distance here.


## 5 Anaphoric reference and conversational interaction

Like exophoric demonstratives, anaphoric demonstratives refer to entities in the speech situation. However, they do so indirectly, by referring to a noun phrase in the surrounding discourse:

(17)

**Table:** 

[*Der*	*Anwalt*]_i_	*sprach*	*mit*	[*einem*	*Klienten*]_j_.	*Da*	*er*_i_ */der*_j_	*nicht*	
the	lawyer	talked	with	a	client	since	he/this_one	not	

*viel*	*Zeit*	*hatte,*	*vereinbarten*	*sie*	*ein*	*weiteres*	*Gespräch*	*nächste*	
much	time	had	agreed_on	they	a	further	conversation	next	

*Woche.*									
week									
‘The lawyer talked to a client. Since he didn’t have much time, they agreed to have another meeting next week.’									
(Diessel 1999: 96)									(German, IE/Germanic)

Unlike the personal pronoun *er*, the demonstrative pronoun *der* can only be coreferential with *ein Klient* ‘a client’: the demonstrative pronoun indicates a topic shift (Diessel 1999: 96). We also use the term anaphoric for demonstratives referring to (the interpretation of) larger bodies of text:34This is part of what Diessel (1999: 100–105) calls the discourse deictic use of demonstratives. However, we only include references to propositions here (e.g. *That’s false*), not references to illocutions (e.g. *That’s a lie*). The latter are more like exophoric reference for us, since illocutions have properties like phonological form, which give them a place in the real world.

(18)

**Table:** 

[*Sales have been going up since 2019*]_i_. [*This trend*]_i_ *is the result of a growing interest*…

An intuitive hypothesis concerning the difference between *this* and *that* in these contexts would be that *this* refers to referents that are more proximal, in terms of either distance (length of text between antecedent and anaphor) or focus (*this* referring to newer or more important information; cf. Strauss 2002). Experimental work of Çokal et al. (2014) found no evidence for this, however, and other studies have found that proximal demonstratives are more likely than distal demonstratives to refer to antecedents further back in the text, contrary to what such an intuitive hypothesis would predict (Maes et al. 2022). Yet another problem for this intuitive hypothesis is that anaphoric this and that cannot be used contrastively (19b) while their exophoric counterparts can (19a):

(19)

**Table:** 

a.	*I don’t want this one, give me that one*. (distinguishing two objects on a table)
b.	*I went Christmas shopping and bought a t-shirt_*i*_ *and a CD*_j_ *; that*_i_ *is for Kim, and this*_j_ *is for Paul.*
	(Stirling and Huddleston 2002: 1506)

All in all, there does not seem to be any positive evidence for exploitation of the actual distance, that is, properties of the relation between deictic expression and referent. We return to this issue in the conclusion.

However, the choice between a proximal and distal demonstrative does seem to be conditioned by Addressee involvement: the relations between the referent and the interlocutors. Evidence for this comes from corpus linguistics, in particular when it comes to the comparison of different corpora. According to Peeters et al. (2021: 421), the ratio of proximal versus distal anaphoric demonstratives varies widely as a function of text or discourse genre. The strongest preference for proximal demonstratives is found in scientific, expository literature, whereas interactional spoken discourse shows a preference for distal demonstratives. Distal demonstratives are also preferred in written news stories, but to a lesser extent. Peeters et al. already recognize that the main difference between these types of corpora is the type of interaction between Speaker (writer) and Addressee (“news corpora … in which information is clearly targeted towards the news item’s consumer”; Peeters et al. 2021: 421). We can make this more concrete with the notion of Shared Discourse Space. In spoken dialogue, there is continuous feedback from the Addressee to the Speaker. As a result, the Speaker can be relatively sure that the Addressee follows along and is attentively involved in the discourse. Thus, as with exophoric demonstratives, the use of the distal form here suggests reference to an element of the Shared Discourse Space between Speaker and Addressee. The same is true for news stories, which are written to be easily accessible by a wide audience. They are somewhat like monologues: there is no feedback from the Addressee, but the content is adjusted so that the Speaker can assume that the Addressee can follow. This is not true for scientific literature, where the high information density and wide variety of reader backgrounds seem to prevent the writer from assuming a large Shared Discourse Space with the Addressee. This means that scientific authors will more frequently assume that their readers do not share in the author’s Personal Discourse Space, and hence use proximal demonstratives more frequently.35This suggestion generates falsifiable hypotheses that can be tested against other types of corpora. For instance, we would expect spoken monologues to show a slightly lower preference for distal demonstratives than interactional discourse, because there is less feedback from the Addressee. Also the fact that evaluative discourse shows a lesser preference for distal demonstratives than regular interactional discourse (Peeters et al. 2021: 421) can be explained this way, since evaluations are inherently personal and not in the Shared Discourse Space. On the other end of the spectrum we would expect to find more distal demonstratives in oral scientific discourse (e.g., conference presentations) than in scientific literature.


These hypotheses have been confirmed for written text in a corpus study by Maes et al. (2022), on the basis of written news stories, Wikipedia articles, and product reviews: “Text genres can be seen as carrying a default assumed psychological distance between writer and referents” (Maes et al. 2022: 26). An anonymous reviewer remarks that these correlations between genre and demonstrative variance can also be related to other factors, such as register (*that* being less formal). We agree that more work needs to be done in this area. However, at this point an explanation based on Addressee involvement strikes us as more economical. Addressee involvement can be related to the [±distal] feature that demonstratives obviously carry, and is independently needed to explain the data described in Sections 2–4. Since the same notion can also explain the genre effect observed by Maes et al. (2022), there is, lacking evidence to the contrary, no need to overcomplicate things by adding a register feature to the analysis.36Note also that if we were to explain the genre effect with register, it is not clear yet why *that* would be associated with more informal registers, since there does not seem to be anything [+distal] about informality (we might expect the contrary!). However, if we have an independent explanation for the genre effect based on Addressee involvement, the correlation with register is a simple consequence of the genre effect. We conclude with Peeters et al. (2021: 422) that the choice between anaphoric *this* and *that* is conditioned primarily by the question whether the referent is “in close psychological proximity to the knowledgeable speaker or writer” or in “the shared space between speaker and addressee”. The proximal/distal distinction in anaphoric demonstratives is therefore primarily recycled to mark Addressee involvement.

## 6 Conclusions

### 6.1 Generalizing over sentential and nominal reference

We have proposed a unified analysis of the recycling of the proximal/distal distinction between the demonstratives *this* and *that* in terms of actual distance (the “distance” between deictic expression and referent) and Addressee involvement (the “distance” between Speaker and referent). This theory is also able to explain the correlation of *this* and *that* with direct and indirect speech reports, respectively, as well as the alternation between *that* (or a parallel finite complementizer) and a zero complementizer in a variety of contexts. These abstract distances are interpreted in different ways depending on the type of referent, as shown in Table 1, reproduced from the introduction.

The four types of environments discussed above have been categorized according to two binary properties here. First, our deictic elements refer to either information content or entities. We studied information content in Sections 2 (direct and indirect speech) and 3 (presuppositions), and entities in Sections 4 and 5 (exophoric and anaphoric demonstratives, respectively). Second, the well-known distinction between exophoric and anaphoric demonstratives for reference to entities generalizes to information content, where it distinguishes utterances from their meaning. Both exophoric demonstratives and speech reports refer directly to concrete things in the world (entities and utterances), whereas anaphoric demonstratives and the complementizers referring to the Shared Discourse Space refer only indirectly (to entities via linguistic antecedents, and to information content through a mental model of the discourse state).

There are two gaps in Table 1. First, actual distance (the “distance” between referent and deictic expression) does not seem to be used in anaphoric reference. We can understand why this is the case in the following way. In both exophoric and anaphoric reference there is a direct link between the referent and the Speaker, namely in the cognitive model of the Speaker. This allows the proximal/distal distinction to be recycled to mark Addressee involvement. But a direct link between the referent and the deictic expression, which is needed to describe actual distance, only exists in exophoric reference: in anaphoric reference, the link is indirect, through an intermediate linguistic entity. The fact that this link is indirect seems to make it difficult to interpret the distance expressed by the proximal/distal element in terms of the relation between referent and deictic expression in these cases, and therefore there is no actual distance there.

Second, proximal elements appear to be incompatible with anaphoric reference to information content (presuppositions): the complementizer *that* alternates with a zero complementizer rather than with a complementizer based on proximal *this*. This gap has already been explained in Section 3.4: using a [−distal] element in this type of reference would suggest that the Speaker refers to informational content that is new to the Addressee (because it is not in the Shared Discourse Space) without introducing it (because anaphoric reference is used). Such use of language would be incompatible with cooperative conversation. In other words, the analysis presented here explains why finite complementizers are so rarely derived from proximal demonstratives.

What unifies the interpretation of Addressee involvement in all four contexts is the fact that the referent is presented as accessible to, or tracked by, the Addressee. Depending on the context, this may have some further implications. This is particularly visible with demonstrative *that*, as shown in Section 4. In these contexts, *that* is in opposition not only with *this* (which would explicitly mark the referent as in the Personal Discourse Space of the Speaker) but also with the definite article *the*. The latter has no [±distal] feature, but can still be used in contexts where the referent is mentally accessible to the Addressee. Consider the following contrast:

(20)

**Table:** 

a.	*Could you pass me the hammer?*
b.	*Could you pass me that hammer?*

Example (20a) can be used in a context where the Addressee is either already tracking the hammer in their Personal Discourse Space, or can easily identify it – that is, *the* already implies Addressee involvement. As a result, the meaning of *that* becomes more marked: excluding pointing contexts where actual distance is promoted, (20b) is most natural in situations where the Addressee is already tracking the hammer, not in situations where the hammer is only identifiable. We thus see that the interpretation of the Addressee involvement marked by [+distal] *that* becomes more marked when it enters into an opposition with *the*. Such an opposition is not available for the complementizer *that*. As a result, the interpretation of Addressee involvement is simpler in this environment, and is confined to referring to an element of the Shared Discourse Space.

### 6.2 Related work

In this paper we have sought to bring together a number of well-known and much studied phenomena in a single theory. We do not have space here to review the full history of scholarship of all these phenomena individually. However, work on some of these issues has, without relating them to the other phenomena, reached similar conclusions to ours, and therefore deserves discussion here.

In particular, there is a long history of work on so-called optional *that* in English, some of which has been referred to in Section 3.2. Of these, Yaguchi (2001) presents an analysis that is quite close to ours: her paper “elucidates the function of the non-deictic *that* by considering how the residual meaning of the demonstrative *that* is still in effect […] and what underpins the presence or absence of the non-deictic *that* from a cognitive perspective” (Yaguchi 2001: 1126). While Yaguchi reaches similar descriptive generalizations based on similar data, we believe the analysis needs refinement. In particular, Yaguchi (2001: 1127) describes the complementizer *that* as “non-deictic”, while she claims at the same time that it also preserves the function of the demonstrative to “deictically point”. It is unclear how the two can be reconciled. Furthermore, Yaguchi (2001: 1127) takes a leap by assuming that “non-deictic” *that* has to do with truth: “the use of demonstratives implicitly encodes the speaker’s presupposition that the hearer can identify the entity to which the speaker refers […] By the same token, non-deictic *that* […] signals that the speaker presupposes the contents of the complement clause to be referential, in other words, to contain true or valid information, whose validity can be proven by evidence.” We agree that *that* is referential in both uses and that this can entail presupposition, but stress that it is perfectly possible to refer to things that are not true or valid. This shows, for instance, in (7) above, where *that* is used to acknowledge an implicit question of the Addressee:

(7)

a.

**Table:** 

*I thought you might need some help.*
(Bolinger 1972: 58)

b.

**Table:** 

*I thought that you might need some help.*
(Bolinger 1972: 58)

For this reason we have analyzed *that* using references to a Shared Discourse Space which, unlike the Common Ground, does not only contain presupposed propositions but also other information content, such as questions or rejected propositions, and entities. Yaguchi (2001: 1137–1139) discusses verbs like *think*, *believe*, and *guess*, which do not presuppose their complement, but does not use referentiality to explain the use of *that* with these verbs. Instead, the distance expressed by *that* would mark the greater amount of evidence and analytic thinking used to come to the conclusion stated in the complement. Yaguchi does not specify, however, how speakers choose between these different factors (referentiality and amount of evidence) when interpreting an instance of *that*. Furthermore, Yaguchi’s approach is problematic for verbs like *doubt*, which suggest that the Speaker favors presupposing the negation of the complement. Even if the use of *that* in *I doubt that* *P* has to do with the amount of evidence, it has to do with the amount of evidence for ¬*P* rather than for *P*. By contrast, in our account we analyze all these different cases as involving referentiality. For example, the use of *that* in *I doubt that* *P* reflects that the question whether *P* is the case is tracked by both Speaker and Addressee.

Dor’s (2005) position on optional *that* is also quite similar to ours: he suggests that “the predicates which can embed the bare clause, without the complementizer, are those which entail that a cognitive agent (in the majority of cases, their subject) has made an epistemic claim concerning the truth of the proposition denoted by the embedded clause” (Dor 2005: 347). This improves on Yaguchi (2001) since it accounts for verbs like *doubt*, though we would still widen the scope a bit to involve reference to questions (for which no truth claim has been made) as well. Furthermore, Dor (2005) is primarily descriptive and does not seek to explain why the use of *that* is related to truth claims. In our view (as in that of Bolinger 1972; Yaguchi 2001) this can be explained as a type of reference, and thus connected to the demonstrative *that*.

This brings us to related work on the similarities between demonstratives and finite complementizers highlighted in Table 1 above. Roberts and Roussou (2003: 111–116) dismantle a number of arguments for the supposed synchronic homophony of demonstrative and complementizer *that*, which is the basis for much of what we are doing here. Kayne (2014) argues that the complementizer *that* is still a demonstrative, but one that does not require “pointing”. He also addresses the question why *this* is not a complementizer, providing an explanation based on a first person feature as opposed to our [±distal]. This is compatible with our analysis if first person is seen as an interpretation of [−distal]. Most recently, Ritter and Wiltschko (2019) and Colasanti and Wiltschko (2019) have argued for a nominal Speech Act structure dominating the DP layer. As is well known, Speech Act structure on the CP level is used to mark the relationships between the propositional content and the Speaker and Addressee, thus formalizing the differences between declaratives, exclamatives, interrogatives, and other sentence types. On the DP level, the Speech Act structure would be used to express the relationships of the interlocutors and the described entity – in particular whether it is discourse-old or discourse-new. This formalization is readily applicable to the observations we have discussed in the present article.

### 6.3 Final remarks

By way of conclusion we want to discuss three final points. First, we wish to point out that paying attention to the fact that the two abstract distances are recycled in different ways depending on the type of reference allows us to resolve some apparent paradoxes. For instance, recall that Cheshire (1996) argued that the exophoric demonstrative *that* can express empathy with the Addressee:

(15a)

**Table:** 

*How’s that throat?*
(Lakoff 1974 via Cheshire 1996: 376)

On the other hand, Storms (1966) suggested that in the context of a witness interrogation, sentences without *that* are used “to put the witness at her ease and at the same time to set an unsuspected trap” (Storms 1966: 263). Thus, the demonstrative *that* in (15a) would engage with the Addressee, whereas it is the absence of the complementizer that does this for Storms (1966). By fleshing out what Addressee involvement really means in these different types of environments, the paradox can be resolved: Cheshire (1996) is talking about reference to entities, where the distal demonstrative establishes joint attention and hence empathy; Storms (1966) is talking about information content where Addressee involvement concerns the Common Ground, and hence the establishment of facts. In this way, Addressee involvement is a useful generalization from which other categories, such as empathy (Cheshire 1996) or “relating to knowledge” (Wierzbicka 1988) can be derived.

Second, a unified analysis of demonstratives and complementizers allows us to explain why *that* introduces finite complements rather than non-finite ones. Tsoulas (1996: 298) points out that the finite/non-finite distinction in clausal complementation can be better described in terms of “definite” and “indefinite” propositions. A proposition is definite when it uses a “definite” tense, that is, a tense that specifies a precise temporal point. In this sense, finite complements are “definite” and infinitival complements are “indefinite”; the latter can by their nature not be situated precisely in space. The selection of a tensed complement by the complementizer *that* can be derived from its demonstrative nature: it references the precise temporal point. In other words, the fact that the complementizer *that* takes finite complements is fully analogous to the fact that demonstratives are necessarily definite (in the common sense): both require their referent to be situated in space and time.

Finally, we might wonder where the relativizer *that* fits in Table 1 above. Its position is clearly in the lower left quadrant for anaphoric reference to entities. However, note that there is no [−distal] relativizer (*the book* *this/*that is on the table here*), which matches with the complementizer *that* in the lower right quadrant (anaphoric reference to information content). We can explain the lack of a proximal relativizer in the same way as we explained the lack of proximal reference to information content: since the referent/antecedent is mentioned in the immediately surrounding context, it is necessarily in the Shared Discourse Space and can therefore not be referred to by a proximal element. Therefore, although the relativizer *that* stands in the lower left quadrant, Addressee involvement is interpreted not as interaction/empathy with the Addressee (as with other anaphoric reference to entities) but using Shared Discourse Space (as with reference to information content). We thus find the distinction between overt and zero relativizers to be similar to that between overt and zero complementizers. For example, (21a) is uttered out of the blue by a detective sergeant to a responding officer, and the Speaker does not expect there to have been anything unusual. The relative clause thus does not have any grounding in space-time or previous discourse, and *that* can be omitted. On the other hand, suppose a customer is looking through the racks in a clothing store. The salesclerk may then ask (21b), where a zero complementizer would be odd: the fact that the customer is looking for something is presupposed. There is a well-defined set of items from which the answer can be drawn (all the clothes in the racks), in contrast to the open-ended nature of (21a).

(21)

**Table:** 

a.	*There was nothing unusual Ø caught your eye when you came in?*
	(*Inspector Morse*, season 7, episode 1)
b.	*Was there anything that/?Ø caught your eye while browsing through the racks?*

In this paper we have analyzed a number of high-frequency uses of the proximal/distal distinction, but our discussion has not been comprehensive. It is expected that actual distance and Addressee involvement can be interpreted differently in other contexts. What we do commit to is the position that the proximal/distal distinction is interpreted in terms of the distance between Speaker and referent (Addressee involvement) and/or deictic expression and referent (actual distance). In this way, the present paper provides an instrumentarium for further analysis of other kinds of reference.

## References

[j_ling-2022-0178_ref_001] Auer Peter (1998). Zwischen Parataxe und Hypotaxe: ,Abhängige Hauptsätze‘ im gesprochenen und geschriebenen Deutsch [Between parataxis and hypotaxis: ‘Dependent main clauses’ in spoken and written German]. *Zeitschrift für Germanistische Linguistik*.

[j_ling-2022-0178_ref_002] Axel-Tober Katrin (2017). The development of the declarative complementizer in German. *Language*.

[j_ling-2022-0178_ref_003] Bate Danny L (In preparation). Where did *that* come from? The distribution and development of neutral finite complementizers in Indo-European. ..

[j_ling-2022-0178_ref_004] Beal Joan, Honey John, Nixon Graham (1988). Goodbye to all ‘that’? The history and present behaviour of optional ‘that’. *An historic tongue: Studies in English linguistics in memory of Barbara Strang*.

[j_ling-2022-0178_ref_005] Biberauer Theresa (2017). Factors 2 and 3: A principled approach. *Cambridge Occasional Papers in Linguistics*.

[j_ling-2022-0178_ref_006] Bolinger Dwight (1972). *That’s that* (Janua Linguarum 155).

[j_ling-2022-0178_ref_007] Brasoveanu Adrian, Farkas Donka F., Alboiu Gabriela, Avram Andrei A., Avram Larisa, Isac Daniela (2007). Say reports, assertion events and meaning dimensions. *Pitar Moş: A building with a view. Papers in honour of Alexandra Cornilescu*.

[j_ling-2022-0178_ref_008] Büring Daniel (2003). On D-trees, beans, and B-accents. *Linguistics and Philosophy*.

[j_ling-2022-0178_ref_009] Chen Rong (1990). English demonstratives: A case of semantic expansion. *Language Sciences*.

[j_ling-2022-0178_ref_010] Cheshire Jenny (1996). That jacksprat: An interactional perspective on English *that*. *Journal of Pragmatics*.

[j_ling-2022-0178_ref_011] Churchland Paul M (1986). Some reductive strategies in cognitive neurobiology. *Mind*.

[j_ling-2022-0178_ref_012] Clark Herbert H (1996). *Using language*.

[j_ling-2022-0178_ref_013] Clark Herbert H., Gerrig Richard J. (1990). Quotations as demonstrations. *Language*.

[j_ling-2022-0178_ref_014] Çokal Derya, Sturt Patrick, Ferreira Fernanda (2014). Deixis: *This* and *that* in written narrative discourse. *Discourse Processes*.

[j_ling-2022-0178_ref_015] Colasanti Valentina, Wiltschko Martina (2019). Spatial and discourse deixis and the speech act structure of nominals. *Proceedings of the 2019 annual conference of the Canadian Linguistics Association*.

[j_ling-2022-0178_ref_016] Corne Chris (1995). Nana k nana, nana k napa: The paratactic and hypotactic relative clauses of Réunion Creole. *Journal of Pidgin and Creole Languages*.

[j_ling-2022-0178_ref_017] Coventry Kenny R., Valdés Berenice, Castillo Alejandro, Guijarro-Fuentes Pedro (2008). Language within your reach: Near–far perceptual space and spatial demonstratives. *Cognition*.

[j_ling-2022-0178_ref_018] Cruschina Silvio, Remberger Eva-Maria, Hummel Martin, Valera Salvador (2017). Before the complementizer: Adverb types and root clause modification. *Adjective adverb interfaces in Romance* (Linguistics Today 242).

[j_ling-2022-0178_ref_019] Dehé Nicole, Wichmann Anne (2010). Sentence-initial *I think (that)* and *I believe (that)*: Prosodic evidence for uses as main clause, comment clause and discourse marker. *Studies in Language*.

[j_ling-2022-0178_ref_020] Delsing Lars-Olof (2010). Exclamatives in Scandinavian. *Studia Linguistica*.

[j_ling-2022-0178_ref_021] Diessel Holger (1999). *Demonstratives. Form, function, and grammaticalization* (Typological Studies in Language 42).

[j_ling-2022-0178_ref_022] Diessel Holger, Tomasello Michael (2001). The acquisition of finite complement clauses in English: A corpus-based analysis. *Cognitive Linguistics*.

[j_ling-2022-0178_ref_023] Dor Daniel (2005). Toward a semantic account of *that*-deletion in English. *Linguistics*.

[j_ling-2022-0178_ref_024] Elsness Johan (1984). *That* or zero? A look at the choice of object clause connective in a corpus of American English. *English Studies*.

[j_ling-2022-0178_ref_025] Etxepare Ricardo (1997). *The grammatical representation of speech events*.

[j_ling-2022-0178_ref_026] Etxepare Ricardo (2010). From hearsay evidentiality to samesaying relations. *Lingua*.

[j_ling-2022-0178_ref_027] Evans Nicholas, Nikolaeva Irina (2007). Insubordination and its uses. *Finiteness: Theoretical and empirical foundations*.

[j_ling-2022-0178_ref_028] Farkas Donka F., Bruce Kim B. (2010). On reacting to assertions and polar questions. *Journal of Semantics*.

[j_ling-2022-0178_ref_029] Ginzburg Jonathan, Seligman Jerry, Westerståhl Dag (1996). Dynamics and the semantics of dialogue. *Language, logic, and computation*, vol. 1 (CSLI Lecture Notes).

[j_ling-2022-0178_ref_030] Gutiérrez-Rexach Javier, D’hulst Yves, Rooryck Johan, Schroten Jan (2001). Spanish exclamatives and the interpretation of the left periphery. *Romance languages and linguistic theory 1999* (Current Issues in Linguistic Theory 221).

[j_ling-2022-0178_ref_031] Hernanz M. Lluïsa, Eguren Luis, Soriano Olga Fernández (2007). From polarity to modality. Some (a)symmetries between *bien* and *sí* in Spanish. *Coreference, modality, and focus: Studies on the syntax-semantics interface* (Linguistics Today 111).

[j_ling-2022-0178_ref_032] Hill Virginia, Aelbrecht Lobke, Haegeman Liliane, Nye Rachel (2012). A main clause complementizer. *Main clause phenomena: New horizons* (Linguistics Today 190).

[j_ling-2022-0178_ref_033] Hooper Joan B., Thompson Sandra A. (1973). On the applicability of root transformations. *Linguistic Inquiry*.

[j_ling-2022-0178_ref_034] Huddleston Rodney, Pullum Geoffrey K. (2002). *The Cambridge grammar of the English language*.

[j_ling-2022-0178_ref_035] Jouitteau Mélanie, Chand Vineeta, Kelleher Ann, Rodríguez Angelo J., Schmeiser Benjamin (2004). Gestures as expletives: Multichannel syntax. *WCCFL 23 proceedings. Proceedings of the 23rd West Coast Conference on Formal Linguistics*.

[j_ling-2022-0178_ref_036] Jungbluth Konstanze, Lenz Friedrich (2003). Deictics in the conversational dyad: Findings in Spanish and some cross-linguistic outlines. *Deictic conceptualisation of space, time and person* (Pragmatics & Beyond New Series 112).

[j_ling-2022-0178_ref_037] Kajzer-Wietrzny Marta, Russo Mariachiara, Bendazzoli Claudio, Defrancq Bart (2018). Interpretese vs. non-native language use: The case of optional *that*. *Making way in corpus-based interpreting studies* (New Frontiers in Translation Studies).

[j_ling-2022-0178_ref_038] Kaltenböck Gunther (2006). ‘*… That* is the question’: Complementizer omission in extraposed *that*-clauses. *English Language and Linguistics*.

[j_ling-2022-0178_ref_039] Kaltenböck Gunther (2009). Initial *I think*: Main or comment clause?. *Discourse and Interaction*.

[j_ling-2022-0178_ref_040] Kayne Richard S, Svenonius Peter (2014). Why isn’t *this* a complementizer?. *Functional structure from top to toe*.

[j_ling-2022-0178_ref_041] Kirsner Robert S, Givón Talmy (1979). Deixis in discourse: An exploratory quantitative study of the Modern Dutch demonstrative adjectives. *Discourse and syntax* (Syntax and Semantics 12).

[j_ling-2022-0178_ref_042] Kocher Anna (2022). *Complementizers on edge: On the boundaries between syntax and pragmatics in Ibero-Romance* (Open Romance Linguistics 1).

[j_ling-2022-0178_ref_043] Krapova Iliyana (2010). Bulgarian relative and factive clauses with an invariant complementizer. *Lingua*.

[j_ling-2022-0178_ref_044] Kratzer Angelika, Rizzi Luigi, Pires de Oliveira Roberta, Emmel Ina, Stedile Monica Deitos, Pires de Oliveira Roberta, Emmel Ina, Quarezemin Sandra (2020). Informal formal conversation on syntax-semantics. *Brazilian Portuguese, syntax and semantics. 20 Years of Núcleo de Estudos Gramaticais*.

[j_ling-2022-0178_ref_045] Lakoff Robin, La Galy Michael W., Fox Robert A., Bruck Anthony (1974). Remarks on ‘this’ and ‘that’. *Papers from the tenth regional meeting of the Chicago Linguistics Society*.

[j_ling-2022-0178_ref_046] Lasnik Howard, Saito Mamoru, Dobrin Lise M., Nichols Lynn, Rodriguez Rosa M. (1991). On the subject of infinitives. *Papers from the 27th regional meeting of the Chicago Linguistic Society. Part one: The general session*.

[j_ling-2022-0178_ref_047] Ledgeway Adam, Benincà Paola, Munaro Nicola (2011). Subject licensing in CP: The Neapolitan double-subject construction. *Mapping the left periphery: The cartography of syntactic structures*.

[j_ling-2022-0178_ref_048] Lewis David (1969). *Convention: A philosophical study*.

[j_ling-2022-0178_ref_049] Maes Alfons, Krahmer Emiel, Peeters David (2022). Explaining variance in writers’ use of demonstratives: A corpus study demonstrating the importance of discourse genre. *Glossa: A Journal of General Linguistics*.

[j_ling-2022-0178_ref_050] Michaelis Laura, Haspelmath Martin, König Ekkehard, Oesterreicher Wulf, Raible Wolfgang (2001). Exclamative constructions. *Language typology and language universals*.

[j_ling-2022-0178_ref_051] Peeters David, Krahmer Emiel, Maes Alfons (2021). A conceptual framework for the study of demonstrative reference. *Psychonomic Bulletin & Review*.

[j_ling-2022-0178_ref_052] Pérez Carlos Muñoz, Verdecchia Matías (2022). Information-based “island effects” in Spanish clausal doubling. ..

[j_ling-2022-0178_ref_053] Pesetsky David (1994). *Zero syntax: Experiencers and cascades*.

[j_ling-2022-0178_ref_054] Poschmann Claudia (2008). All declarative questions are attributive?. *Belgian Journal of Linguistics*.

[j_ling-2022-0178_ref_055] Quirk Randolph, Greenbaum Sidney, Leech Geoffrey, Svartvik Jan (1985). *A comprehensive grammar of the English language*.

[j_ling-2022-0178_ref_056] Rissanen Matti, Aijmer Karin, Altenberg Bengt (1991). On the history of *that*/zero as object clause links in English. *English corpus linguistics. Studies in honour of Jan Svartvik*.

[j_ling-2022-0178_ref_057] Ritter Elizabeth, Wiltschko Martina (2019). Nominal speech act structure: Evidence from the structural deficiency of impersonal pronouns. *Canadian Journal of Linguistics/Revue canadienne de linguistique*.

[j_ling-2022-0178_ref_058] Rizzi Luigi, Haegeman Liliane (1997). The fine structure of the left periphery. *Elements of grammar* (Kluwer International Handbooks of Linguistics 1).

[j_ling-2022-0178_ref_059] Roberts Craige (2012 [1996]). Information structure in discourse: Towards an integrated formal theory of pragmatics. *Semantics & Pragmatics*.

[j_ling-2022-0178_ref_060] Roberts Ian, Roussou Anna (2003). *Syntactic change. A minimalist aproach to grammaticalization* (Cambridge Studies in Linguistics 100).

[j_ling-2022-0178_ref_061] Rooryck Johan, Koster Jan, van Riemsdijk Henk (2003). The morphosyntactic structure of articles and pronouns in Dutch. *Germania et alia: A linguistic webschrift for Hans den Besten*.

[j_ling-2022-0178_ref_062] Rooryck Johan, Bağrıaçık Metin, Breitbarth Anne, De Clercq Karen (2019). ‘Recycling’ evidentiality: A research program. *Mapping linguistic data. Essays in honour of Liliane Haegeman*.

[j_ling-2022-0178_ref_063] Rosenbaum Peter Steven (1965). *The grammar of English predicate complement constructions*.

[j_ling-2022-0178_ref_064] Ross John Robert, Jacobs Roderick A., Rosenbaum Peter S. (1970). On declarative sentences. *Readings in English transformational grammar*.

[j_ling-2022-0178_ref_065] Sornicola Rosanna, Benincà Paola, Cinque Guglielmo, de Mauro Tullio, Vincent Nigel (1996). Alcune strutture con pronome espletivo nei dialetti italiani meridionali [Some structures with expletive pronouns in Southern Italian dialects]. *Italiano e dialetti nel tempo. Saggi di grammatica per Giolio C. Lepschy* [*Italian and dialects over time. Essays in grammar for Giolio C. Lepschy*].

[j_ling-2022-0178_ref_066] Speas Peggy, Tenny Carol, Maria Di Sciullo Anna (2003). Configurational properties of point of view roles. *Asymmetry in grammar*. Vol. 1: *Syntax and semantics* (Linguistics Today 57).

[j_ling-2022-0178_ref_067] Stalnaker Robert, Cole Peter (1978). Assertion. *Pragmatics* (Syntax and Semantics 9).

[j_ling-2022-0178_ref_068] Stalnaker Robert (2002). Common Ground. *Linguistics and Philosophy*.

[j_ling-2022-0178_ref_069] Stirling Lesley, Huddleston Rodney, Huddleston Rodney, Pullum Geoffrey K. (2002). Deixis and anaphora. *The Cambridge grammar of the English language*.

[j_ling-2022-0178_ref_070] Storms Godfrid (1966). *That*-clauses in Modern English. *English Studies*.

[j_ling-2022-0178_ref_071] Strauss Susan (2002). *This, that*, and *it* in spoken American English: A demonstrative system of gradient focus. *Language Sciences*.

[j_ling-2022-0178_ref_072] Thompson Sandra A (2002). “Object complements” and conversation. Towards a realistic account. *Studies in Language*.

[j_ling-2022-0178_ref_073] Thompson Sandra A, Mulac Anthony (1991). The discourse conditions for the use of the complementizer *that* in conversational English. *Journal of Pragmatics*.

[j_ling-2022-0178_ref_074] Trotzke Andreas, Villalba Xavier, Trotzke Andreas, Villalba Xavier (2021). Expressive insubordination: A cross-linguistic study on *that*-exclamatives. *Expressive meaning across linguistic levels and frameworks*.

[j_ling-2022-0178_ref_075] Tsoulas George, Zagona Karen T. (1996). The nature of the subjunctive and the formal grammar of obviation. *Grammatical theory and Romance languages: Selected papers from the 25th Linguistic Symposium on Romance Languages (LSRL XXV), Seattle, 2–4 March 1995* (Current Issues in Linguistic Theory 133).

[j_ling-2022-0178_ref_076] Visan Florentina (2000). The nature and status of exclamatives in Chinese. ..

[j_ling-2022-0178_ref_077] Weinert Regina (2012). Complement clauses in spoken German and English: Syntax, deixis and discourse-pragmatics. *Folia Linguistica*.

[j_ling-2022-0178_ref_078] Wierzbicka Anna (1988). *The semantics of grammar* (Studies in Language Companion Series 18).

[j_ling-2022-0178_ref_079] Yaguchi Michiko (2001). The function of the non-deictic *that* in English. *Journal of Pragmatics*.

[j_ling-2022-0178_ref_080] Zanuttini Raffaella, Portner Paul (2003). Exclamative clauses: At the syntax-semantics interface. *Language*.

[j_ling-2022-0178_ref_081] Zevakhina Natalia (2013). Syntactic strategies of exclamatives. Eesti ja soome-ugri keeleteaduse ajakiri. *Journal of Estonian and Finno-Ugric Linguistics*.

